# ST-Segment Elevation Myocardial Infarction After Pegfilgrastim: Case Report and Review of Mechanistic Considerations

**DOI:** 10.1016/j.jaccas.2025.106046

**Published:** 2025-12-17

**Authors:** Collin Goetze, Henryk Dreger, Cheng-Ying Chiu

**Affiliations:** a Department of Cardiology, Angiology and Intensive Care Medicine, Deutsches Herzzentrum der Charité, Campus Virchow-Klinikum, Berlin, Germany; b Charité–Universitätsmedizin Berlin, Corporate Member of Freie Universität Berlin and Humboldt-Universität zu Berlin, Berlin, Germany; c Deutsches Zentrum für Herzkreislaufforschung (DHZK), Berlin, Germany

**Keywords:** acute coronary syndrome, cancer, complication, myocardial infarction, percutaneous coronary intervention

## Abstract

**Background:**

Granulocyte-colony stimulating factors (G-CSFs) are widely used to prevent chemotherapy-induced neutropenia, but they have been linked to coronary neovascularization and prothrombotic effects.

**Case Report:**

A 62-year-old man with small cell lung cancer developed an acute posterior ST-segment elevation myocardial infarction (STEMI) 1 day after receiving 6 mg of pegfilgrastim during his second chemo-immunotherapy cycle. Coronary angiography revealed an occluded right coronary artery without atherosclerosis. Aspiration thrombectomy significantly reduced thrombus burden, restoring TIMI flow grade 3. Initial blood tests showed leukocytosis (white blood cells: 82.13 × 10^9^/L) and thrombocytosis (platelets: 773 × 10^9^/L), which normalized at discharge. Transesophageal echocardiography excluded embolic sources, and hyperviscosity syndrome was considered.

**Discussion:**

Marked leukocytosis and thrombocytosis after pegfilgrastim plausibly triggered acute myocardial infarction via leukostasis/hyperviscosity, endothelial activation, neutrophil extracellular traps, and platelet reactivity. To our knowledge, this first reported STEMI after pegfilgrastim warrants vigilance for leukocytosis-related thromboembolic complications in oncology patients.

**Take-Home-Messages:**

G-CSF–induced leukocytosis should be considered as a rare cause of acute myocardial infarction in patients without significant cardiovascular history. Thrombectomy remains a valuable adjunctive tool.

## Central Illustration

**Figure:**
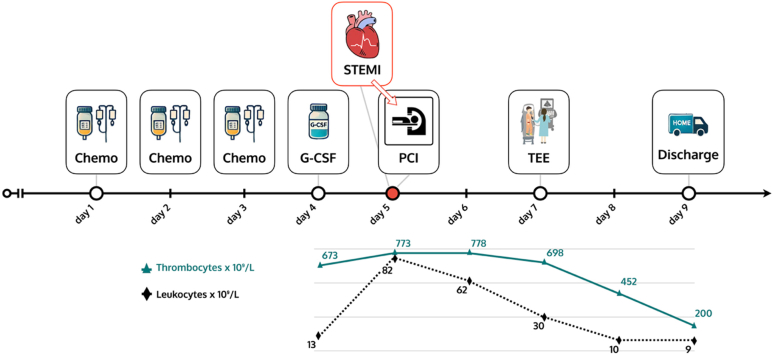

**Visual Summary:**
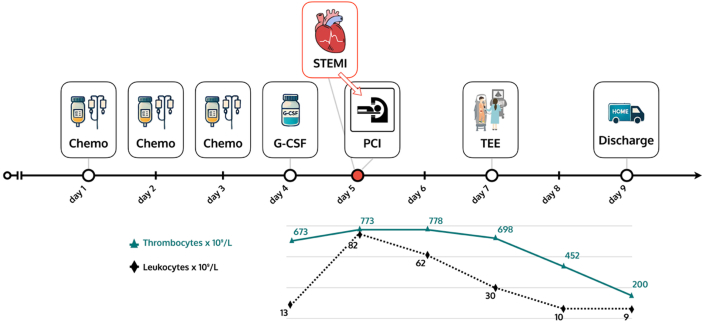
Timeline Illustrating the Clinical Course and Laboratory Dynamics G-CSF = granulocyte-colony stimulating factor; PCI = percutaneous coronary intervention; STEMI = ST-segment elevation myocardial infarction; TEE = transesophageal echocardiography.

## History of Presentation

**Figure 1:**
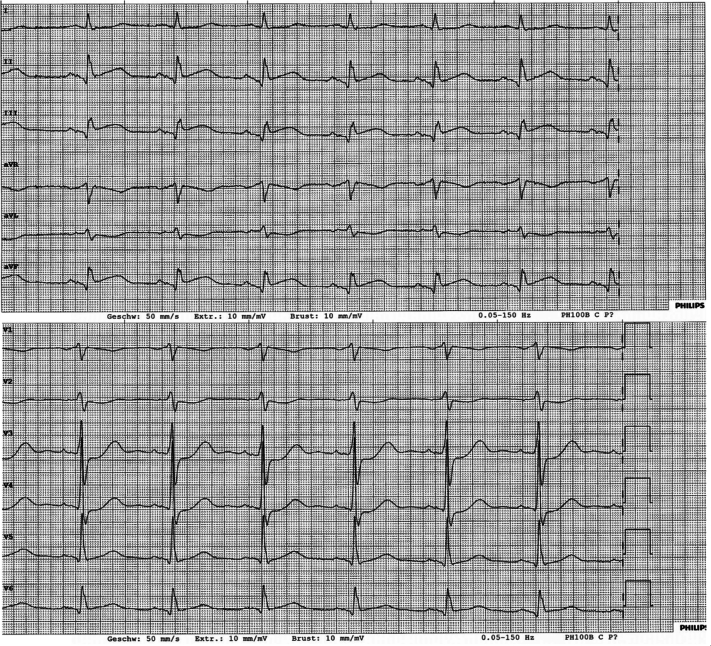
Twelve-Lead ECG at Time of Presentation ECG shows sinus rhythm with ST-segment elevation in leads II, III, and aVF and ST-segment depression in leads aVL and V_2_ to V_4_. ECG = electrocardiogram.

A 62-year-old man presented with typical angina pectoris at the emergency department by ambulance. The electrocardiogram at admission showed ST-segment elevations in the inferior leads (Figure 1), and the patient was directly transferred to the cardiac catheterization laboratory.

**Take-Home Messages**

• G-CSF therapy, although an effective treatment for neutropenia, may in rare cases lead to leukocytosis-associated thromboembolic events, warranting careful monitoring in cancer patients.
• This first documented case of STEMI after pegfilgrastim underscores the importance of recognizing and managing potential cardiovascular complications in vulnerable populations, with thrombectomy as a valuable adjunctive tool.

## Past Medical History

The patient had been discharged from the department of pulmonology the day before, where he received the second cycle of systemic chemo-immunotherapy for metastatic small cell neuroendocrine lung carcinoma. The chemo-immunotherapy regimen included carboplatin, etoposide, and atezolizumab. Additionally, 6 mg of pegfilgrastim had been administered subcutaneously on day 4, which was the day before the presentation in our emergency department. Laboratory tests at the time of pegfilgrastim administration showed a slightly elevated white blood cell (WBC) and platelet count (WBCs: 13 × 10^9^/L, platelets: 673 × 10^9^/L).

The patient had no cardiac history except for paroxysmal atrial fibrillation and arterial hypertension. With a CHA_2_DS_2_-VA score of 1, anticoagulation had been omitted. Prior chest computed tomography scans performed for lung cancer staging did not reveal any calcified coronary plaques, thus no formal coronary calcium scoring had been performed.

## Differential Diagnosis

Differential diagnosis included acute coronary syndrome from plaque rupture or erosion, thromboembolism or coronary spasm, as well as immune checkpoint inhibitor–associated pericarditis and myocarditis.

## Investigations

**Figure 2:**
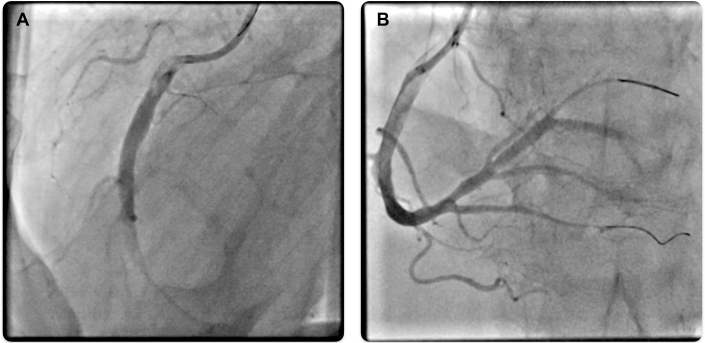
Coronary Angiography (A) Coronary angiogram showing 99% occlusion of the RCA. (B) Coronary angiogram after thrombus aspiration in the mid-RCA, RPL, and PDA using the CAT RX aspiration catheter (Penumbra) and stent implantation in the RPL with TIMI flow grade 3. PDA = posterior descending artery; RCA = right coronary artery; RPL = right posterolateral branch.

Immediate cardiac catheterization was performed. Coronary angiography revealed an acute embolic occlusion of the right coronary artery (Figure 2A). Laboratory tests on admission revealed significantly elevated WBC (82.13 × 10^9^/L) and platelet counts (773 × 10^9^/L).

## Management

Successful retrieval of a macroscopically white thrombus from the right coronary artery was performed with CAT RX aspiration catheter (Penumbra). Given the ongoing high thrombus burden, balloon dilation in the posterior descending artery and the right posterolateral branch was performed, followed by additional thrombus aspiration. Because of a suspected vascular dissection in the right posterolateral branch, a 3.0 × 15 mm stent was implanted. Subsequently, a very good primary outcome with a TIMI flow grade 3 was observed (Figure 2B). The left coronary artery was unremarkable.

Transesophageal echocardiography was performed and ruled out intracardiac thrombus or patent foramen ovale as embolic causes.

The patient had not received granulocyte-colony stimulating factor (G-CSF) during the first cycle of chemo-immunotherapy, and his WBC counts before were within normal range, so the observed leukocytosis was likely due to pegfilgrastim administration. Over time, the significantly elevated WBC count fell, showing 62 × 10^9^/L on day 1 post-STEMI, 30 × 10^9^/L on day 2, and returning to the normal range at 9.6 × 10^9^/L on day 3. His platelet count fell but remained elevated, with levels at 773 × 10^9^/L on admission, 778 × 10^9^/L on day 1, 698 × 10^9^/L on day 2, and 452 × 10^9^/L on day 3.

## Outcome

The patient's symptoms regressed rapidly during his hospital stay. Both WBC and platelet counts were within normal range at discharge. He was discharged home in good health.

## Discussion

G-CSF and its pegylated form, pegfilgrastim, are used to prevent chemotherapy-related neutropenia. By expanding neutrophils, G-CSF lowers infection risk and supports uninterrupted chemotherapy.^1^ However, the administration of G-CSF is potentially associated with adverse effects. Among the less common but serious complications are hematologic abnormalities such as leukocytosis,^1^^,^^2^ which may pose risks for thrombotic events.

Healthy stem cell donors receiving G-CSF have not demonstrated a significantly increased risk of thromboembolic events after the administration of G-CSF compared with the general population.^2^ However, an increased rate of cardiac events such as in-stent restenosis when administering G-CSF after percutaneous coronary intervention^3^ has been observed in high-risk cardiac patients, possibly explained by proinflammatory properties inducing plaque destabilization.^4^^,^^5^ In addition, case reports and small case series have described a temporal association of myocardial infarction (including non-STEMI after filgrastim),^6^ limb ischemia,^7^ and other ischemic complications^7^ with G-CSF administration, suggesting a prothrombotic signal. In the current case, onset of acute myocardial infarction (AMI) occurred after G-CSF administration—a temporal link that warrants further investigation.

G-CSF exerts multiple effects on the cardiovascular system, among which the effects on inflammation and coagulation are the most notable with respect to ischemic complications. Leukocytosis secondary to G-CSF administration can precipitate leukostasis and hyperviscosity, with microvascular obstruction.^2^^,^^8^ Studies have shown that the release of proinflammatory cytokines (eg, IL-1β, TNF-α) after G-CSF has been associated with endothelial activation and heightened adhesiveness as well as leukocyte infiltration with increased tissue factor expression and formation of neutrophil extracellular traps (NETs) driving the thrombosis-inflammation circuit. In addition, augmentation of functional platelet activity resulting in augmentation of platelet aggregation has been described.^4^^,^^5^^,^^7^^,^^9^ Complementing these human data, in vivo murine work has demonstrated G-CSF–triggered, inflammation-associated cardiac thrombosis in susceptible states (eg, iron-loaded myocardium), which is mitigated by statin therapy.^9^ Taken together, G-CSF–induced leukocytosis and G-CSF–driven inflammation can promote cardiovascular complications, such as cardiac thrombosis.

The concomitant administration of immune checkpoint inhibitors such as atezolizumab may further augment proinflammatory signaling, potentially enhancing the risk of vascular complications when combined with G-CSF. This is biologically plausible, as both drug classes act through pathways that could create a synergistic prothrombotic environment in a milieu of cancer-related inflammation and myelosuppressive therapy, thereby increasing the vulnerability to acute ischemic events.

AMI is usually linked to traditional risk factors such as hypertension, hyperlipidemia, diabetes, and obesity.^6^ It is notable that our patient had no significant cardiac history aside from paroxysmal atrial fibrillation and arterial hypertension. The absence of radiographic evidence of coronary atherosclerosis on prior computed tomography imaging strengthens the likelihood that the acute event was precipitated by treatment-related factors rather than progression of pre-existing obstructive coronary artery disease. The presence of atrial fibrillation indicates an elevated thromboembolic risk, especially in the absence of anticoagulation therapy, and the probability that the embolic STEMI was induced by atrial fibrillation is greater than in a comparable individual without atrial fibrillation. However, a CHA_2_DS_2_-VA score of 1 did not warrant anticoagulation, as the estimated annual thromboembolic risk of 1.9%^10^ is considered below the treatment threshold according to current guideline-based risk stratification. Our patient's low-risk cardiac profile underscores the unusual nature of the AMI in the context of pegfilgrastim administration and raises important considerations regarding the safety profile of G-CSF in relation to potential cardiac complications.

## Conclusions

To our knowledge, this is the first reported case of STEMI directly after G-CSF/pegfilgrastim administration. This case highlights a potential link between G-CSF treatment and acute myocardial infarction in cancer patients without significant cardiovascular history. Although G-CSF remains a valuable therapy for managing chemotherapy-induced neutropenia, clinicians should be vigilant for rare thromboembolic complications, particularly in patients with predisposing factors. Further research is necessary to better understand these risks and define vulnerable populations. Given the rarity of such events, systematic pharmacovigilance reporting and the establishment of registry-based datasets are essential to better define the incidence, risk factors, and outcomes of G-CSF–associated cardiovascular complications.

## Funding Support and Author Disclosures

The authors have reported that they have no relationships relevant to the contents of this paper to disclose.
